# Supplementary material for: Asynchronous magnetic resonance elastography: Shear wave speed reconstruction using noise correlation of incoherent waves
Source: Magn Reson Med. 2022 Oct 27;89(3):990–1001. doi: 10.1002/mrm.29502 (PMC9792433; doi:10.1002/mrm.29502)
Supplement: Supplementary file 1 — DATA S1 MATLAB code used to generate the numerical simulations of Figure 1 [file MRM-89-990-s002.zip › k-Wave/helpfiles/pstdElastic2D.html]

pstdElastic2D :: Functions (k-Wave)


# pstdElastic2D

2D time-domain simulation of elastic wave propagation.

## Syntax

```
sensor_data = pstdElastic2D(kgrid, medium, source, sensor)
sensor_data = pstdElastic2D(kgrid, medium, source, sensor, ...)
```

## Description

`pstdElastic2D` simulates the time-domain propagation of elastic waves through a two-dimensional homogeneous or heterogeneous medium given four input structures: `kgrid`, `medium`, `source`, and `sensor`. The computation is based on a pseudospectral time domain model which accounts for viscoelastic absorption and heterogeneous material parameters. At each time-step (defined by `kgrid.dt` and `kgrid.Nt` or `kgrid.t_array`), the wavefield parameters at the positions defined by `sensor.mask` are recorded and stored. If `kgrid.t_array` is set to `'auto'`, this array is automatically generated using the `makeTime` method of the `kWaveGrid` class. An anisotropic absorbing boundary layer called a perfectly matched layer (PML) is implemented to prevent waves that leave one side of the domain being reintroduced from the opposite side (a consequence of using the FFT to compute the spatial derivatives in the wave equation). This allows infinite domain simulations to be computed using small computational grids.

An initial pressure distribution can be specified by assigning a matrix of pressure values the same size as the computational grid to `source.p0`. This is then assigned to the normal components of the stress within the simulation function. A time varying stress source can similarly be specified by assigning a binary matrix (i.e., a matrix of 1's and 0's with the same dimensions as the computational grid) to `source.s_mask` where the 1's represent the grid points that form part of the source. The time varying input signals are then assigned to `source.sxx`, `source.syy`, and `source.sxy`. These can be a single time series (in which case it is applied to all source elements), or a matrix of time series following the source elements using MATLAB's standard column-wise linear matrix index ordering. A time varying velocity source can be specified in an analogous fashion, where the source location is specified by `source.u_mask`, and the time varying input velocity is assigned to `source.ux` and `source.uy`.

The field values are returned as arrays of time series at the sensor locations defined by `sensor.mask`. This can be defined in three different ways. (1) As a binary matrix (i.e., a matrix of 1's and 0's with the same dimensions as the computational grid) representing the grid points within the computational grid that will collect the data. (2) As the grid coordinates of two opposing corners of a rectangle in the form [x1; y1; x2; y2]. This is equivalent to using a binary sensor mask covering the same region, however, the output is indexed differently as discussed below. (3) As a series of Cartesian coordinates within the grid which specify the location of the pressure values stored at each time step. If the Cartesian coordinates don't exactly match the coordinates of a grid point, the output values are calculated via interpolation. The Cartesian points must be given as a 2 by N matrix corresponding to the x and y positions, respectively, where the Cartesian origin is assumed to be in the center of the grid. If no output is required, the `sensor` input can be replaced with an empty array `[]`.

If `sensor.mask` is given as a set of Cartesian coordinates, the computed `sensor_data` is returned in the same order. If `sensor.mask` is given as a binary matrix, `sensor_data` is returned using MATLAB's standard column-wise linear matrix index ordering. In both cases, the recorded data is indexed as `sensor_data(sensor_point_index, time_index)`. For a binary sensor mask, the field values at a particular time can be restored to the sensor positions within the computation grid using `unmaskSensorData`. If `sensor.mask` is given as a list of opposing corners of a rectangle, the recorded data is indexed as `sensor_data(rect_index).p(x_index, y_index, time_index)`, where `x_index` and `y_index` correspond to the grid index within the rectangle, and `rect_index` corresponds to the number of rectangles if more than one is specified.

By default, the recorded acoustic pressure field is passed directly to the output `sensor_data`. However, other acoustic parameters can also be recorded by setting `sensor.record` to a cell array of the form `{'p', 'u', 'p_max', ...}`. For example, both the particle velocity and the acoustic pressure can be returned by setting `sensor.record = {'p', 'u'}`. If `sensor.record` is given, the output `sensor_data` is returned as a structure with the different outputs appended as structure fields. For example, if `sensor.record = {'p', 'p_final', 'p_max', 'u'}`, the output would contain fields `sensor_data.p`, `sensor_data.p_final`, `sensor_data.p_max`, `sensor_data.ux`, and `sensor_data.uy`. Most of the output parameters are recorded at the given sensor positions and are indexed as `sensor_data.field(sensor_point_index, time_index)` or `sensor_data(rect_index).field(x_index, y_index, time_index)` if using a sensor mask defined as opposing rectangular corners. The exceptions are the averaged quantities (`'p_max'`, `'p_rms'`, `'u_max'`, `'p_rms'`, `'I_avg'`), the 'all' quantities (`'p_max_all'`, `'p_min_all'`, `'u_max_all'`, `'u_min_all'`), and the final quantities (`'p_final'`, `'u_final'`). The averaged quantities are indexed as `sensor_data.p_max(sensor_point_index)` or `sensor_data(rect_index).p_max(x_index, y_index)` if using rectangular corners, while the final and 'all' quantities are returned over the entire grid and are always indexed as `sensor_data.p_final(nx, ny)`, regardless of the type of sensor mask.

`pstdElastic2D` may also be used for time reversal image reconstruction by assigning the time varying pressure recorded over an arbitrary sensor surface to the input field `sensor.time_reversal_boundary_data`. This data is then enforced in time reversed order as a time varying Dirichlet boundary condition over the sensor surface given by `sensor.mask`. The boundary data must be indexed as `sensor.time_reversal_boundary_data(sensor_point_index, time_index)`. If `sensor.mask` is given as a set of Cartesian coordinates, the boundary data must be given in the same order. An equivalent binary sensor mask (computed using nearest neighbour interpolation) is then used to place the pressure values into the computational grid at each time step. If `sensor.mask` is given as a binary matrix of sensor points, the boundary data must be ordered using MATLAB's standard column-wise linear matrix indexing. If no additional inputs are required, the `source` input can be replaced with an empty array `[]`.

## Inputs

The minimum fields that must be assigned to run an initial value problem (for example, a photoacoustic forward simulation) are marked with a \*.

|  |  |
| --- | --- |
| `kgrid*` | k-Wave grid object returned by `kWaveGrid` containing Cartesian and k-space grid fields |
| `kgrid.t_array*` | evenly spaced array of time values [s] (set to `'auto'` by `kWaveGrid`) |
|  |  |
| `medium.sound_speed_compression*` | compressional sound speed distribution within the acoustic medium [m/s] |
| `medium.sound_speed_shear*` | shear sound speed distribution within the acoustic medium [m/s] |
| `medium.density*` | density distribution within the acoustic medium [kg/m^3] |
| `medium.alpha_coeff_compression` | absorption coefficient for compressional waves [dB/(MHz^2 cm)] |
| `medium.alpha_coeff_shear` | absorption coefficient for shear waves [dB/(MHz^2 cm)] |
|  |  |
| `source.p0*` | initial pressure within the acoustic medium |
| `source.sxx` | time varying stress at each of the source positions given by `source.s_mask` |
| `source.syy` | time varying stress at each of the source positions given by `source.s_mask` |
| `source.sxy` | time varying stress at each of the source positions given by `source.s_mask` |
| `source.s_mask` | binary matrix specifying the positions of the time varying stress source distributions |
| `source.s_mode` | optional input to control whether the input stress is injected as a mass source or enforced as a dirichlet boundary condition; valid inputs are `'additive'` (the default) or `'dirichlet'` |
| `source.ux` | time varying particle velocity in the x-direction at each of the source positions given by `source.u_mask` |
| `source.uy` | time varying particle velocity in the y-direction at each of the source positions given by `source.u_mask` |
| `source.u_mask` | binary matrix specifying the positions of the time varying particle velocity distribution |
| `source.u_mode` | optional input to control whether the input velocity is applied as a force source or enforced as a dirichlet boundary condition; valid inputs are `'additive'` (the default) or `'dirichlet'` |
|  |  |
| `sensor.mask*` | binary matrix or a set of Cartesian points where the pressure is recorded at each time-step |
| `sensor.record` | cell array of the acoustic parameters to record in the form `sensor.record = {'p', 'u', ...}`; valid inputs are:    `'p'` (acoustic pressure)    `'p_max'` (maximum pressure)    `'p_min'` (minimum pressure)    `'p_rms'` (RMS pressure)    `'p_final'` (final pressure field at all grid points)    `'p_max_all'` (maximum pressure at all grid points)    `'p_min_all'` (minimum pressure at all grid points)    `'u'` (particle velocity)    `'u_max'` (maximum particle velocity)    `'u_min'` (minimum particle velocity)    `'u_rms'` (RMS particle velocity)    `'u_final'` (final particle velocity field at all grid points)    `'u_max_all'` (maximum particle velocity at all grid points)    `'u_min_all'` (minimum particle velocity at all grid points)    `'u_non_staggered'` (particle velocity on non-staggered grid)    `'u_split_field'` (particle velocity on non-staggered grid split into compressional and shear components)    `'I'` (time varying acoustic intensity)    `'I_avg'` (average acoustic intensity)   NOTE: the acoustic pressure outputs are calculated from the normal stress via p = -(sxx + syy)/2 |
| `sensor.record_start_index` | time index at which the sensor should start recording the data specified by `sensor.record` (default = 1) |
| `sensor.time_reversal_boundary_data` | time varying pressure enforced as a Dirichlet boundary condition over `sensor.mask` |

**Note**  For a heterogeneous medium, `medium.sound_speed_compression`, `medium.sound_speed_shear`, and `medium.density` must be given in matrix form with the same dimensions as `kgrid`. For a homogeneous medium, these can be given as scalar values.

## Optional Inputs

Optional 'string', value pairs that may be used to modify the default computational settings.

| Input | Valid Settings | Default | Description |
| --- | --- | --- | --- |
| `'CartInterp'` | `'linear'` `'nearest'` | `'linear'` | Interpolation mode used to extract the pressure when a Cartesian sensor mask is given. If set to `'nearest'` and more than one Cartesian point maps to the same grid point, duplicated data points are discarded and `sensor_data` will be returned with less points than that specified by `sensor.mask`. |
| `'CreateLog'` | *(Boolean scalar)* | `false` | Boolean controlling whether the command line output is saved using the diary function with a date and time stamped filename. |
| `'DataCast'` | *(string of data type)* | `'off'` | String input of the data type that variables are cast to before computation. For example, setting to `'single'` will speed up the computation time (due to the improved efficiency of `fftn` and `ifftn` for this data type) at the expense of a loss in precision. This variable is also useful for utilising GPU parallelisation through libraries such as the Parallel Computing Toolbox by setting `'DataCast'` to `'gpuArray-single'`. |
| `'DataRecast'` | *(Boolean scalar)* | `false` | Boolean controlling whether the output data is cast back to double precision. If set to false, `sensor_data` will be returned in the data format set using the `'DataCast'` option. |
| `'DisplayMask'` | *(binary matrix)* or | `sensor.mask` | Binary matrix overlaid onto the animated simulation display. Elements set to 1 within the display mask are set to black within the display. |
| `'MovieArgs'` | *(string cell array)* | `{}` | Settings for `VideoWriter`. Parameters must be given as {'param', value, ...} pairs within a cell array (default = {}), where 'param' corresponds to a writable property of a VideoWriter object. |
| `'MovieName'` | *(string)* | `'date-time-kspaceFirstOrder2D'` | Name of the movie produced when `'RecordMovie'` is set to `true`. |
| `'MovieProfile'` | *(string)* | `'Uncompressed AVI'` | Profile input passed to `VideoWriter`. |
| `'PlotFreq'` | *(integer numeric scalar)* | `10` | The number of iterations which must pass before the simulation plot is updated. |
| `'PlotPML'` | *(Boolean scalar)* | `true` | Boolean controlling whether the perfectly matched layer is shown in the simulation plots. If set to `false`, the PML is not displayed. |
| `'PlotScale'` | *(numeric four element vector)* or `'auto'` | `[-1, 1, -1, 1]` | `[sii_min, sii_max, sij_min, sij_max]` values used to control the scaling for `imagesc` (visualisation), where `sii` is the plot scale for the normal stress, and `sij` is the plot scale for the shear stress. If set to `'auto'`, a symmetric plot scale is chosen automatically for each plot frame. |
| `'PlotSim'` | *(Boolean scalar)* | `true` | Boolean controlling whether the simulation iterations are progressively plotted. |
| `'PMLAlpha'` | *(numeric scalar or three element vector)* | `2` | Absorption within the perfectly matched layer in Nepers per grid point. |
| `'PMLInside'` | *(Boolean scalar)* | `true` | Boolean controlling whether the perfectly matched layer is inside or outside the grid. If set to `false`, the input grids are enlarged by `PMLSize` before running the simulation. |
| `'PMLSize'` | *(integer numeric scalar or three element vector)* | `10` | Size of the perfectly matched layer in grid points. By default, the PML is added evenly to all sides of the grid, however, both `PMLSize` and `PMLAlpha` can be given as two element arrays to specify the x and y properties, respectively. To remove the PML, set the appropriate `PMLAlpha` to zero rather than forcing the PML to be of zero size. |
| `'RecordMovie'` | *(Boolean scalar)* | `false` | Boolean controlling whether the displayed image frames are captured and stored as a movie using `VideoWriter` (default = false). |
| `'Smooth'` | *(Boolean scalar or three element vector)* | `[true, false, false]` | Boolean controlling whether `source.p0`, `medium.sound_speed`, and `medium.density` are smoothed using `smooth` before computation. `'Smooth'` can either be given as a single Boolean value or as a 3 element array to control the smoothing of `source.p0`, `medium.sound_speed`, and `medium.density`, independently. |

## Outputs

If `sensor.record` is not defined by the user:

|  |  |
| --- | --- |
| `sensor_data` | time varying pressure recorded at the sensor positions given by `sensor.mask` |

If `sensor.record` is defined by the user:

|  |  |
| --- | --- |
| `sensor_data.p` | time varying pressure recorded at the sensor positions given by `sensor.mask` (returned if `'p'` is set) |
| `sensor_data.p_max` | maximum pressure recorded at the sensor positions given by `sensor.mask` (returned if `'p_max'` is set) |
| `sensor_data.p_min` | minimum pressure recorded at the sensor positions given by `sensor.mask` (returned if `'p_min'` is set) |
| `sensor_data.p_rms` | rms of the time varying pressure recorded at the sensor positions given by `sensor.mask` (returned if `'p_rms'` is set) |
| `sensor_data.p_final` | final pressure field at all grid points within the domain (returned if `'p_final'` is set) |
| `sensor_data.p_max_all` | maximum pressure recorded at all grid points within the domain (returned if `'p_max_all'` is set) |
| `sensor_data.p_min_all` | minimum pressure recorded at all grid points within the domain (returned if `'p_min_all'` is set) |
| `sensor_data.ux` | time varying particle velocity in the x-direction recorded at the sensor positions given by `sensor.mask` (returned if `'u'` is set) |
| `sensor_data.uy` | time varying particle velocity in the y-direction recorded at the sensor positions given by `sensor.mask` (returned if `'u'` is set) |
| `sensor_data.ux_max` | maximum particle velocity in the x-direction recorded at the sensor positions given by `sensor.mask` (returned if `'u_max'` is set) |
| `sensor_data.uy_max` | maximum particle velocity in the y-direction recorded at the sensor positions given by `sensor.mask` (returned if `'u_max'` is set) |
| `sensor_data.ux_min` | minimum particle velocity in the x-direction recorded at the sensor positions given by `sensor.mask` (returned if `'u_min'` is set) |
| `sensor_data.uy_min` | minimum particle velocity in the y-direction recorded at the sensor positions given by `sensor.mask` (returned if `'u_min'` is set) |
| `sensor_data.ux_rms` | rms of the time varying particle velocity in the x-direction recorded at the sensor positions given by `sensor.mask` (returned if `'u_rms'` is set) |
| `sensor_data.uy_rms` | rms of the time varying particle velocity in the y-direction recorded at the sensor positions given by `sensor.mask` (returned if `'u_rms'` is set) |
| `sensor_data.ux_final` | final particle velocity field in the x-direction at all grid points within the domain (returned if `'u_final'` is set) |
| `sensor_data.uy_final` | final particle velocity field in the y-direction at all grid points within the domain (returned if `'u_final'` is set) |
| `sensor_data.ux_max_all` | maximum particle velocity in the x-direction recorded at all grid points within the domain (returned if `'u_max_all'` is set) |
| `sensor_data.uy_max_all` | maximum particle velocity in the y-direction recorded at all grid points within the domain (returned if `'u_max_all'` is set) |
| `sensor_data.ux_min_all` | minimum particle velocity in the x-direction recorded at all grid points within the domain (returned if `'u_min_all'` is set) |
| `sensor_data.uy_min_all` | minimum particle velocity in the y-direction recorded at all grid points within the domain (returned if `'u_min_all'` is set) |
| `sensor_data.ux_non_staggered` | time varying particle velocity in the x-direction recorded at the sensor positions given by `sensor.mask` after shifting to the non-staggered grid (returned if `'u_non_staggered'` is set) |
| `sensor_data.uy_non_staggered` | time varying particle velocity in the y-direction recorded at the sensor positions given by `sensor.mask` after shifting to the non-staggered grid (returned if `'u_non_staggered'` is set) |
| `sensor_data.ux_split_p` | compressional component of the time varying particle velocity in the x-direction on the non-staggered grid recorded at the sensor positions given by `sensor.mask` (returned if `'u_split_field'` is set) |
| `sensor_data.ux_split_s` | shear component of the time varying particle velocity in the x-direction on the non-staggered grid recorded at the sensor positions given by `sensor.mask` (returned if `'u_split_field'` is set) |
| `sensor_data.uy_split_p` | compressional component of the time varying particle velocity in the y-direction on the non-staggered grid recorded at the sensor positions given by `sensor.mask` (returned if `'u_split_field'` is set) |
| `sensor_data.uy_split_s` | shear component of the time varying particle velocity in the y-direction on the non-staggered grid recorded at the sensor positions given by `sensor.mask` (returned if `'u_split_field'` is set) |
| `sensor_data.Ix` | time varying acoustic intensity in the x-direction recorded at the sensor positions given by `sensor.mask` (returned if `'I'` is set) |
| `sensor_data.Iy` | time varying acoustic intensity in the y-direction recorded at the sensor positions given by `sensor.mask` (returned if `'I'` is set) |
| `sensor_data.Ix_avg` | average acoustic intensity in the x-direction recorded at the sensor positions given by `sensor.mask` (returned if `'I_avg'` is set) |
| `sensor_data.Iy_avg` | average acoustic intensity in the z-direction recorded at the sensor positions given by `sensor.mask` (returned if `'I_avg'` is set) |

## Examples

- Explosive Source In A Layered Medium
- Plane Wave Absorption
- Shear Waves And Critical Angle Reflection

## See Also

`kspaceFirstOrder2D`, `kWaveGrid`, `pstdElastic3D`
